# Bioavailability and Antidiabetic Activity of Gliclazide-Loaded Cubosomal Nanoparticles

**DOI:** 10.3390/ph14080786

**Published:** 2021-08-09

**Authors:** Mohamed Nasr, Saud Almawash, Ahmed Al Saqr, Alaa Y. Bazeed, Sameh Saber, Heba I. Elagamy

**Affiliations:** 1 Department of Pharmaceutics, Faculty of Pharmacy, Delta University for Science and Technology, Gamasa 35712, Egypt; alaayosf@gmail.com (A.Y.B.); hebaelagamy1985@yahoo.com (H.I.E.); 2 Department of Pharmaceutics and Industrial Pharmacy, Faculty of Pharmacy, Helwan University, Cairo 11790, Egypt; 3 Department of Pharmaceutical Sciences, College of Pharmacy, Shaqra University, Shaqra 15581, Saudi Arabia; salmawash@su.edu.sa; 4 Department of Pharmaceutics, College of Pharmacy, Prince Sattam Bin Abdulaziz University, Al-Kharj 11942, Saudi Arabia; a.alsaqr@psau.edu.sa; 5 Department of Pharmacology, Faculty of Pharmacy, Delta University for Science and Technology, Gamasa 11152, Egypt; sampharm81@gmail.com

**Keywords:** gliclazide, BCS class II drug, cubosomes, bioavailability, antidiabetic activity

## Abstract

In this study, gliclazide-loaded cubosomal particles were prepared for improving the oral bioavailability and antidiabetic activity of gliclazide. Four formulations of gliclazide-loaded cubosomal nanoparticles dispersions were prepared by the emulsification method using four different concentrations of glyceryl monooleate (GMO) and poloxamer 407 (P407) as the stabilizer. The prepared formulations were in vitro and in vivo evaluated. In vitro, the prepared gliclazide-loaded cubosomal dispersions exhibited disaggregated regular poly-angular particles with a nanometer-sized particle range from 220.60 ± 1.39 to 234.00 ± 2.90 nm and entrapped 73.84 ± 3.03 to 88.81 ± 0.94 of gliclazide. In vitro gliclazide release from cubosomal nanoparticles revealed an initially higher drug release during the first 2 h in acidic pH medium; subsequently, a comparatively higher drug release in alkaline medium relative to gliclazide suspension was observed. An in vivo absorption study in rats revealed a two-fold increase in the bioavailability of gliclazide cubosomal formulation relative to plain gliclazide suspension. Moreover, the study of in vivo hypoglycemic activity indicated that a higher percentage reduction in glucose level was observed after the administration of gliclazide cubosomal nanoparticles to rats. In conclusion, gliclazide-loaded cubosomal nanoparticles could be a promising delivery system for improving the oral absorption and antidiabetic activity of gliclazide.

## 1. Introduction

Gliclazide, a second-generation sulfonylurea, is an effective oral hypoglycemic drug adopted to treat non-insulin-dependent diabetes mellitus. Gliclazide is a Biopharmaceutical Classification System (BCS) class II drug [1] with poor dissolution rate [2,3]. Due to its low and pH-dependent solubility [4,5], gliclazide has an irregular and slow absorption rate which can result in large intra- and inter-individual changes in absorption following oral administration [6,7]. Gliclazide is commercially available as conventional fast release or modified release tablets with a variable daily dose ranged from 40 to 80 mg in two divided doses.

Several formulation techniques have been adapted to improve the bioavailability and therapeutic effectiveness of gliclazide such as the preparation of solid dispersion [8,9], inclusion complex with β-cyclodextrin [10], nanocrystals [11], liquid–solid systems [12], solid lipid nanoparticles [13], and self-micro-emulsifying delivery system [14]. The previously mentioned approaches rely on increasing the solubility of gliclazide as a mechanism for bioavailability improvement. In the present study, we are aiming to utilize the potential of cubosomal nanoparticles as a drug delivery system to enhance the oral bioavailability of gliclazide.

Cubosomes, nanostructured particles of bicontinuous cubic liquid crystalline phase, are formulated easily by a hydrating mixture of glyceryl-monooleate and poloxamer 407. The cubic phase produces colloidal and thermodynamically stable particulate dispersions [15,16]. Cubosomes have many benefits, such as high drug encapsulating and loading ability of hydrophilic and hydrophobic active pharmaceutical ingredients (APIs), simple preparation techniques, lipids biodegradability, and both sustained and targeted release of drugs [15,16,17,18].

Cubosomes have been suggested as a promising delivery system for orally administered drugs. In certain cases, cubosomes have been investigated as a drug delivery system for poorly water-soluble drugs to increase oral bioavailability [19]. Previously, cubosomal nanoparticles have been used as a drug delivery system for enhancing the bioavailability of simvastatin as a BCS class II drug [20] and cyclosporine A as a model of BCS IV drug of poor water solubility and poor permeability [21]. Moreover, cubosomal nanoparticles have been recently utilized as a delivery system for the intraperitoneal administration of antidiabetic drug repaglinide [22]. In this study, gliclazide-loaded cubosomal nanoparticles were used as a delivery system for improving the oral bioavailability and antidiabetic activity of gliclazide. In a trial to achieve this objective, gliclazide-loaded cubosomal nanoparticles were fabricated, in vitro and in vivo assessed to evaluate their impact on bioavailability and therapeutic activity of gliclazide.

## 2. Results and Discussion

### 2.1. Particle Size, Polydispersity Index, and Zeta Potential

**Figure 1:**
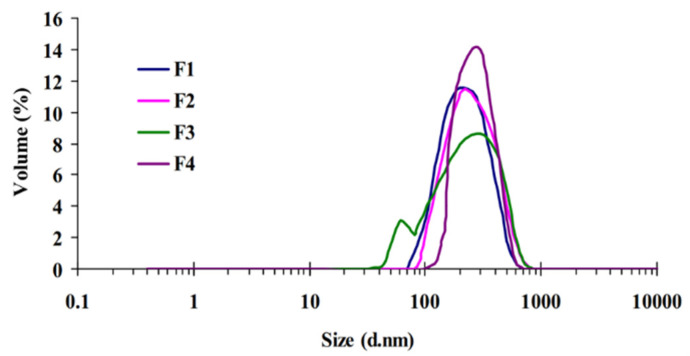
Particle size distribution of gliclazide-loaded nanoparticles.

The mean values of particle size, PDI, and zeta potential of the prepared cubosomes are presented in Table 1. All dispersions showed a narrow monomodal particle size distribution (Figure 1). The obtained mean particle sizes of cubosomal nanoparticles ranged from 220.60 ± 1.39 to 234.00 ± 2.90 nm. There were no significant differences between particle sizes of all formulas; however, a slight increase in the particles size was associated with increasing GMO concentration from 1.25 to 7.50% *w*/*w*. The PDI values were in the range between 0.098 ± 0.017 and 0.172 ± 0.002, indicating acceptable homogeneity of all dispersions. The zeta potential values for all cubosomal formulas were in the range of −19.40 ± 0.80 to −25.30 ± 0.22 mV, indicating a high degree of stability with lower tendency for particles aggregation [23].

### 2.2. Entrapment Efficiency

The obtained values of EE % ranged between 73.84 ± 3.03 and 88.81 ± 0.94 (Table 1) from the total gliclazide content (90–95%). The high EE % of gliclazide in all formulae may be attributed to its highly lipophilic nature. The results revealed a direct correlation between EE % of gliclazide and GMO concentration up to 5%. However, increasing GMO concentration to 7.50% (F4), the EE % did not show any significant change compared to F3.

### 2.3. Differential Scanning Calorimetry (DSC)

**Figure 2:**
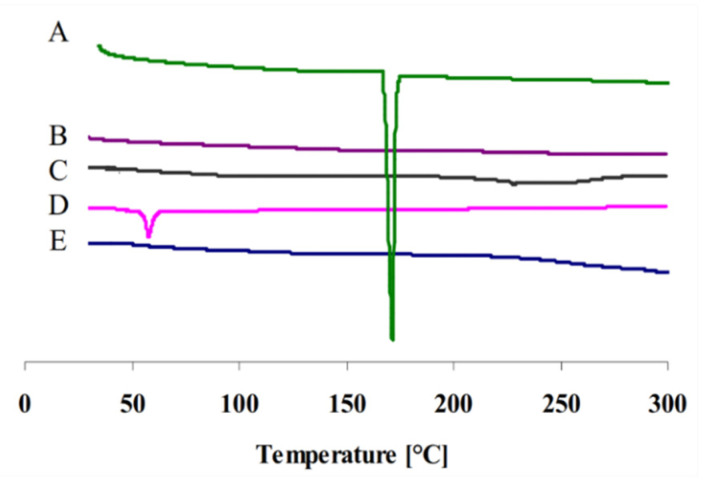
DSC thermograms of plain gliclazide (**A**), gliclazide-loaded cubosomal nanoparticles (**B**), blank cubosomal nanoparticles (**C**), P407 (**D**), and GMO (**E**).

DSC thermograms of plain gliclazide, gliclazide-loaded cubosomal nanoparticles, blank cubosomal nanoparticles, P407, and GMO are presented in Figure 2. Gliclazide powder presented a thermogram with a sharp endothermic peak of 171.82 °C indicating its crystalline nature [24] and P407 showed an endothermic melting peak at 55 °C [25]. However, this peak had been completely vanished in the DSC thermogram of cubosomal nanoparticles, indicating that gliclazide was incorporated into cubosomes either in a non-crystalline state or converted to molecular state.

### 2.4. Morphology of Gliclazide-Loaded Cubosomes

**Figure 3:**
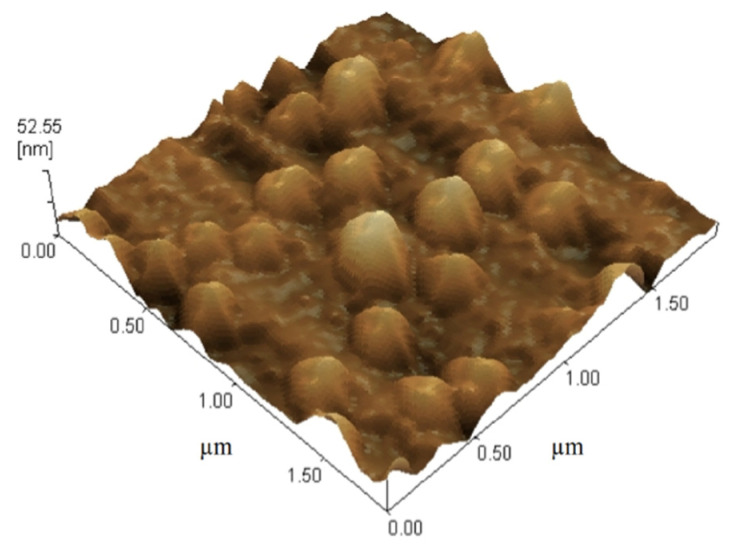
AFM photomicrograph of gliclazide-loaded nanoparticles (F3).

The atomic force microscope AFM image (Figure 3) of gliclazide-cubosomal nanoparticles (F3) showed that the nanoparticles are disaggregated regular poly-angular particles with approximately 52.55 nm of height. In addition, the cubosomal nanoparticles were of nano-size range, which agreed with the measured particles size values.

### 2.5. In Vitro Gliclazide Release

**Figure 4:**
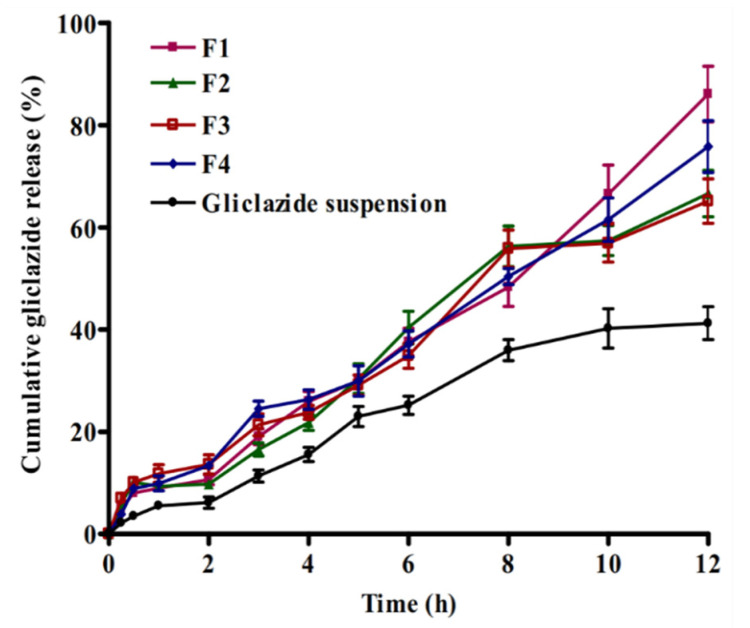
In vitro release profiles (n = 3 ± SD) of gliclazide from different cubosomal nanoparticles formulations (F1–F4) and aqueous gliclazide suspension.

The release profiles of gliclazide-loaded cubosomal nanoparticles are presented in Figure 4. The four formulations show biphasic drug release profiles extended over a period of 12 h. Where during the first two hours, a relatively low initial percentage of gliclazide (9.75 ± 0.98 to 13.62 ± 1.89%) was released from cubosomal formulations. Meanwhile, aqueous gliclazide suspension released a significantly lower percentage compared to cubosomal nanoparticles (*p* < 0.05). The relatively low release of the drug indicates that the major fraction of gliclazide is entrapped within the bilayer of cubosomal particles and only a small fraction of drug is adsorbed on surface of cubosomal nanoparticles [26,27]. Another reason for the lower drug release is the acidic pH of release medium that might counteract the dissolution of gliclazide. This was previously explained based on the ampholytic nature and pH-dependent solubility of gliclazide, where a minimum solubility was recorded in the acidic pH [28,29]. However, when the pH of the medium was changed to 6.8, a significant rise (*p* < 0.05) in the percentage drug released was observed in case of cubosomal nanoparticles in comparison with gliclazide suspension. This may be attributed to nanosized particles of gliclazide-loaded cubosomal nanoparticles [27].

### 2.6. Bioavailability Study

Gliclazide-loaded cubosomal nanoparticles (F3) was selected for in vivo absorption study and antidiabetics activity due to its EE % and reasonable particle size.

**Figure 5:**
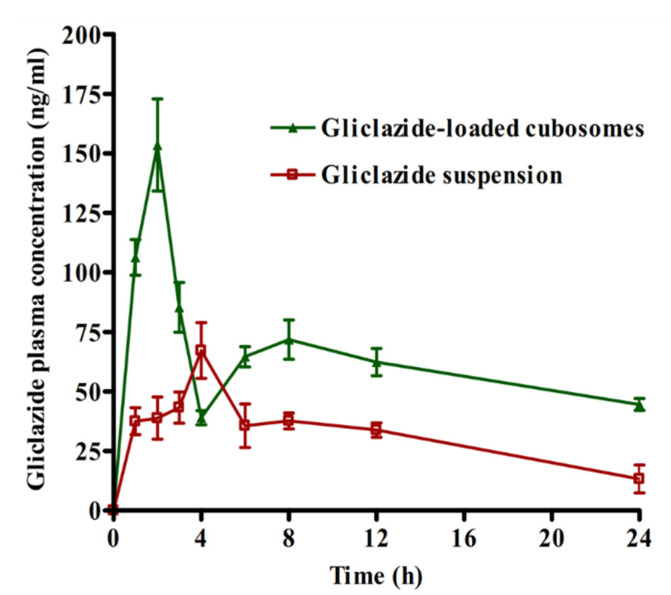
Mean (n = 6 ± SD) gliclazide plasma concentration–time profiles after the administration of a single oral dose (10 mg/kg) of gliclazide-loaded cubosomal nanoparticles and drug suspension to rats.

The rate and extent of drug absorption in terms of C_max_, T_max_, and AUC were utilized to evaluate the bioavailability of gliclazide-loaded cubosomal nanoparticles compared to gliclazide aqueous suspension. Figure 5 shows the mean plasma concentration time profiles of gliclazide after administration of a single oral dose (10 mg/kg) of either gliclazide-loaded cubosomal nanoparticles or gliclazide suspension. In Table 2, the mean values of bioavailability parameters (C_max_, T_max_, and AUC_0-24_) are presented. The statistical analysis of the obtained values revealed significantly (*p* < 0.05) higher values of the maximum plasma concentration and AUC_0-24_ of gliclazide-loaded cubosomes compared to gliclazide aqueous suspension. However, the higher C_max_ value of gliclazide-loaded cubosomal nanoparticles was accompanied by a significant (*p* < 0.05) reduction in T_max_ to 2 h compared to 4 h in case of the gliclazide suspension. This result might be due to the higher percentage of drug released from cubosomal nanoparticles during the first 2 h relative to gliclazide suspension. The mean relative bioavailability of the gliclazide cubosomal nanoparticles was 200.50% ± 10.55. However, a higher bioavailability of gliclazide was previously reported when formulated as solid lipid nanoparticles [13]. The higher bioavailability could be attributed to the structural similarity between the lipid bilayer of cubosomes and cell membrane [30,31], which may facilitate the uptake of cubosomes by endothelial membranes [32]. Moreover, the formation of secondary nanostructures loaded with the drug due to the digestion of GMO in the gastrointestinal tract promotes drug-carrying vesicles to infiltrate the “diffusion layer” [30] and provide more adjacent contact with cell membranes, leading to improvement of drug absorption. In addition, the unique cubosomal structure could minimize degradation of the encapsulated drug in the GIT [33,34].

### 2.7. Evaluation of the Hypoglycemic Activity

**Figure 6:**
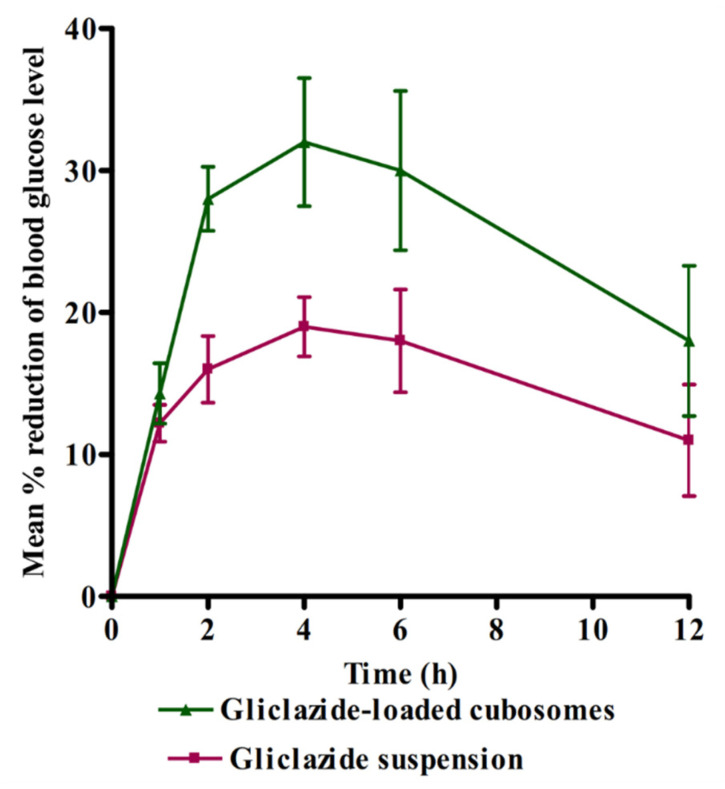
Mean (n = 6 ± SD) % reduction in blood glucose level of diabetic rats after the administration of a single oral dose (10 mg/kg) of gliclazide-loaded cubosomal nanoparticles and drug suspension to diabetic rats.

The influence of cubosomal formulation on the antidiabetic activity of gliclazide was evaluated compared to gliclazide suspension by monitoring the hypoglycemic effect in terms of percentage reduction in blood glucose level versus time profile in experimentally induced diabetic rats after being given a single oral dose of 10 mg/kg of both preparations. The percentage reduction in glucose level–time profiles of the two treatments are presented in Figure 6. It is obvious that the two profiles are different, and a higher percentage reduction in glucose levels in blood was detected after 2, 4, 6, and 12 h post administration of gliclazide cubosomal nanoparticles, with evidence of significantly higher values (*p* < 0.05) of both the mean maximum percentage reduction in blood glucose level and the mean area under the percentage reduction of blood glucose level–time curve in comparison with gliclazide suspension. The obtained results are well correlated with the higher plasma concentration in the cubosomal group as reflected by the higher AUC_0-24_. These results are in good agreement with previous studies that formulated gliclazide in different delivery systems such as solid lipid nanoparticles [13] and mucoadhesive microcapsules [35].

## 3. Materials and Methods

### 3.1. Materials

Gliclazide was granted from Rameda^®^, Cairo, Egypt. Acetonitrile (HPLC grade) was obtained from BDH, Poole, England. Glyceryl monooleate (GMO) was generously provided by Gattefosse, France. Poloxamer 407 (P407) was purchased from Sigma-Aldrich Chemical Company (Milwaukee, WI, USA). Potassium dihydrogen phosphate, disodium hydrogen phosphate (pharmaceutical grade), sodium hydroxide, sodium lauryl sulfate, methanol, phosphoric acid, and hydrochloric acid (analytical grade) were purchased from El Nasr Chemical Company, Cairo, Egypt.

### 3.2. Preparation of Gliclazide-Loaded Cubosomes

Gliclazide-loaded cubosomal nanoparticles were formulated according to Nasr [36]. GMO and poloxamer 407 were heated in a water bath at 60 °C until homogeneity was achieved. Gliclazide was dissolved in methylene chloride, heated to 60 °C, and added to the homogenous mixture of GMO and P407 with continuous stirring. Then, methylene chloride was removed by evaporation in a 40 °C water bath. Deionized water (2 mL) was added drop by drop with continuous stirring to get a gel form. The mixture was left for two days at room temperature, and the remaining amount of the deionized water was added to the resulted gel using vortex mixing. Finally, probe sonication (5 s on and 5 s off) was implemented for 5 min to get the final cubosomal nanoparticles. Four formulations (F1–F4) of gliclazide-loaded cubosomal nanoparticles were prepared using four GMO concentrations of 1.25, 2.5, 5, and 7.5% w/w of the total dispersion and a fixed 10% P407 relative to GMO content. The final gliclazide concentration was 10 mg/g in all formulations [37].

### 3.3. Characterization of Gliclazide-Loaded Cubosomes

#### 3.3.1. Particle Size, Polydispersity Index, and Zeta Potential

The particle analysis (mean hydrodynamic diameter and polydispersity index, PDI) and zeta potential of the different formulated nanoparticles were determined using the Zetasizer Nano series (Nano ZS, Malvern, UK). A 1 mL sample of each formulation was diluted to 20 mL with deionized water and measured at 25 ± 0.5 °C in triplicate.

#### 3.3.2. Entrapment Efficiency Percentage (EE %)

Entrapment efficiency percentage (EE %) was determined by the ultrafiltration centrifugation technique [38] using ultracentrifuge tubes (Amicon, 3000 molecular weight cut-of (MWCO), Millipore, Burlington, MA, USA). Briefly, an aliquot (1 mL) of gliclazide cubosomal nanoparticles was properly diluted and centrifuged at 6000 rpm for 10 min (Sigma 2–16P, Sigma Laborzentrifugen GmbH, Osterode am Harz, Germany). The obtained filtrate was measured spectrophotometrically at 226 nm [39] for free gliclazide (Q_Free_) using a double beam visible spectrophotometer (Jenway, Stafford, UK). The calibration curve was constructed in methanol, 0.1 N HCl and 2 M phosphate buffer (pH 6.8) ranging from 2 to 24 µg/mL. The total gliclazide content presented in 1 mL dispersion (Q_Total_) was determined spectrophotometrically at the same wavelength after dilution with methanol to ensure complete lysis of cubosomes. EE % was calculated using the following equation:

EE % = [(Q_Total_ − Q_Free_)/Q_Total_] × 100.

#### 3.3.3. Morphology of Cubosomal Nanoparticles

The morphology of gliclazide-loaded cubosomes (F3) was observed using a Wet-SPM Scanning Probe atomic force microscope (Shimadzu, Kyoto, Japan). A drop of cubosomal dispersion was adsorbed on freshly cleaved muscovite mica squares, removing excess water by air drying. The sample was mounted in a microscope scanner for viewing and imaging in the non-contact mode at a frequency of 312 kHz and a scan speed of 2 Hz.

#### 3.3.4. Differential Scanning Calorimetry (DSC)

DSC thermograms of gliclazide-loaded cubosomal particles, blank cubosomal particles, plain gliclazide, GMO, and P407 were examined using a DSC-60 differential scanning calorimeter (Shimadzu, Kyoto, Japan). Each sample (5 mg) was heated in an aluminum pan from 30 to 320 °C at a constant rate of 10 °C/min under a nitrogen purge of 30 mL/min. A similar empty pan was used as the reference.

#### 3.3.5. In Vitro Drug Release Study

In vitro gliclazide release from cubosomal nanoparticles was monitored by the dynamic dialysis method [40]. A volume of each cubosomal nanoparticles and drug dispersion in water (equivalent to 10 mg gliclazide) was tightly sealed in a dialysis bag (11,325 MWCO, DO655, Sigma-Aldrich, St. Louis, MO, USA). The study was carried out in a release medium of 125 mL of 0.1 N hydrochloric acid containing 0.25% sodium lauryl sulfate (SLS) for the first two hours, and then the pH is shifted to 6.8 by adding 125 mL of 2 M phosphate buffer. The concentration of SLS in release medium was kept at 0.25% during the entire period of the study to maintain sink conditions. The release medium was stirred at 100 rpm and maintained at 37 ± 0.5 °C. Fresh media was added after aliquots were removed from release medium at predetermined time intervals (0, 0.25, 0.5, 1, 2, 3, 4, 5, 6, 8, 10, and 12 h). The amount of gliclazide released was measured using a UV spectrophotometer at 226 nm [39]. The experiments were conducted in triplicate.

#### 3.3.6. Bioavailability Study

The study protocol was performed after the approval of the Animal Ethics Committee of Faculty of Pharmacy, Delta University for science and technology (Approval number FPDU2121/3). Twelve adult male Wistar rats weighing from 230 to 250 g were used in the experiment. The rats received the same diet regime and were separated into 2 groups. The day before the experiment, all rats were allowed to fast overnight and had only access to water. On the day of experiment, a single oral dose of 10 mg/kg gliclazide aqueous suspension was received by each rat in the first (control) group, while each rat in the second group was given an equivalent dose of cubosomal nanoparticles using an oral feeding tube. Blood samples (0.5 mL) were withdrawn from the lateral tail vein in heparinized tubes at different intervals (0, 1, 2, 3, 4, 6, 8, 12 and 24 h) post-dose administration using a 22 G butterfly needle. Before analysis, blood samples were centrifuged for 10 min at 5000 rpm, and plasma was separated. The drug plasma concentrations were quantified using a reported HPLC method with slight modifications [41]. Briefly, 0.25 mL of plasma was spiked with naftopidil as an internal standard (2 µg in 50 µL), which was followed by the addition of 0.70 mL acetonitrile. The organic layer was evaporated after being separated by centrifugation at 5000 rpm for 10 min. A volume (100 µL) of the mobile phase was added to the residue; then, the resulted solution was filtered, and a volume of 20 µL filtrate was injected at a flow rate of 1.2 mL/min into the HPLC system using Hypersil gold C18, 4.6 × 50 mm, 5.0 μm column (SHIMADZU Corporation, CTO-20A, Kyoto, Japan) with a variable wavelength UV detector (VWD 1260). The mobile phase consisted of (60:40 *v*/*v*) acetonitrile and 20 mm phosphate buffer at pH 4. The method was validated as the calibration showed reasonable linearity in the range of 1–8 µg/mL with correlation coefficient equals 0.992, and the mean percentage recovery was 99.50 ± 1.48, indicating high accuracy of the assay. The RSD % of intra and inter-quality control samples were less than 2%, reflecting a high degree precision of the method. In addition, values of LOD and LOQ were 0.7 and 1 µg/mL, respectively.

The bioavailability of gliclazide-loaded cubosomal nanoparticles (F3) was compared relative to gliclazide suspension in terms of the rate and extent of drug absorption that presented as C_max_, T_max_, and AUC_0-24_. The values of maximum gliclazide concentration in plasma (C_max_, ng/mL) and the time to achieve maximum concentration (T_max_, h) were attained directly from the plasma data of the individual plasma concentration versus time curves. For each rat, the linear trapezoidal rule was used to calculate the values of area under the plasma concentration versus time curve from 0 to 24 h (AUC_0-24_). The calculated values of AUC_0-24_ were used to estimate the relative bioavailability of gliclazide-loaded cubosomal nanoparticles compared to gliclazide aqueous suspension.

#### 3.3.7. Antidiabetic Activity Study

The hypoglycemic activity of gliclazide-loaded cubosomal nanoparticles was evaluated based on the percentage reduction of glucose level in experimentally induced diabetic rats compared to plain gliclazide suspension. The study design and animal manipulation were accepted by the Animal Ethics Committee of Faculty of Pharmacy, Delta University for science and technology (approval number FPDU2121/3). Two groups of albino rats (each of 6) with an average weight of 205.50 ± 5.50 grams were utilized in the study. The induction of experimental diabetes mellitus in rats was achieved by injecting a freshly prepared single intraperitoneal dose of streptozotocin (40 mg/kg body weight). After 48 h, blood samples were drawn to ensure the induction of diabetes. Fasting blood glucose level was determined using a fast take glucometer (Accu-check^®^). The blood glucose level of more than 250 mg/dL was used as an indication of diabetic induction. On the day of the study, the diabetic rats in both groups were allowed to ingest food pellet for half an hour only with free access to water. After two hours, the blood glucose was measured and was an indication for the zero-time glucose level. The gliclazide-loaded cubosomal nanoparticles and gliclazide suspension (10 mg/kg) [13] were administered by oral feeding syringe. Blood samples were withdrawn at certain time intervals (0, 1, 2, 4, 6, and 12 h), and blood glucose level was measured as mentioned before. The percentage reduction of glucose level was calculated based on the measured glucose level at zero-time. The maximum percentage reduction in blood glucose level and time for maximum reduction were determined from the blood glucose time profile of each rat. The area under maximum percentage reduction in blood glucose level–time curve from time 0 to 12 h was calculated by the linear trapezoidal method.

### 3.4. Statistical Analysis

Data were reported as means ± standard deviation (SD). Student’s *t* test (SPSS program; version 12.0) was used to compare the obtained results. A statistically significant difference was considered at *p* value < 0.05.

## 4. Conclusions

Cubosomal nanoparticles containing gliclazide were prepared by the emulsification of GMO in water in the presence of P407 as a stabilizer. The prepared gliclazide-loaded cubosomal nanoparticles exhibited nano-sized particles and entrapped about 80% of gliclazide along with a higher percentage of drug release compared to gliclazide suspension. The gliclazide-loaded nanoparticles successfully enhanced the rate and extent of drug absorption in rats, as evidenced by a two-fold increase in relative bioavailability and a higher area under percentage reduction of glucose level–time curve compared to gliclazide suspension. These results revealed that cubosomal nanoparticles could be a potential carrier for enhancing the oral bioavailability and hypoglycemic activity of gliclazide. However, further stability study is required to ensure the stability of gliclazide in the cubosomal formulation.

## Figures and Tables

**Table 1:** GMO content, particle size, PDI, zeta potential, and EE % of gliclazide-loaded cubosomal nanoparticles.

EE %	Zeta Potential mV	PDI	Particle Size (nm)	GMO % *w*/*w*	Formula
73.84 ± 3.03	−19.40 ± 0.80	0.172 ± 0.002	220.60 ± 1.39	1.25	F1
80.68 ± 1.85	−21.80 ± 0.50	0.142 ± 0.035	225.30 ± 2.40	2.50	F2
88.81 ± 0.94	−24.20 ± 0.91	0.155 ± 0.012	226.50 ± 1.50	5.00	F3
87.42 ± 1.28	−25.30 ± 0.22	0.098 ± 0.017	234.00 ± 2.90	7.50	F4

- Standard deviation was calculated (n = 3).

**Table 2:** Mean values of bioavailability parameters of gliclazide in rats (n = 6 ± SD) after administration of a single oral dose (10 mg/kg) of gliclazide aqueous suspension and equivalent dose of cubosomal nanoparticles (F3).

Bioavailability Parameters	Gliclazide Aqueous Suspension	Gliclazide-Loaded Cubosomal Dispersion (F3)
C_max_ (ng/mL)	67.10 ± 11.73	153.50 ± 19.35 *
T_max_ (h)	4.00	2.00 *
AUC_0-24_ (ng.h/mL)	754.51 ± 67.83	1513.99 ± 196.39 *
Relative bioavailability	-	200.5% ± 10.55

* *p* < 0.05.

## Data Availability

The data presented are available within the article.
